# Practical Notes on Diagnosis and Treatment

**Published:** 1908-01-18

**Authors:** 


### PRACTICAL NOTES ON DIAGNOSIS AND TREATMENT.

**Malignant Endocarditis.**

You have no business to diagnose malignant endocarditis unless the patient has very well-marked physical signs, unless he has a high irregular temperature, and unless he has the phenomena of embolism.—*Dr. Rose Bradford.*

**Functional Hemiplegia.**

When a hemiplegia is accompanied by a profound degree of hemianæsthesia there is a reasonable suspicion that the case is one of functional, and not of organic, disease. This suspicion is strengthened when the anæsthesia involves the mucous membranes, and is further confirmed when the special senses share in the defect. Still more is the conclusion true when the sensory condition extends to loss of all forms of cutaneous sensation, including loss of the sense of pain.—*Dr. C. O. Hawthorne.*

**Cerebro-Spinal Meningitis.**

The removal of fluid by lumbar puncture is distinctly beneficial if there is any indication of intra-cranial pressure.—*Dr. Cautley.*

**Temperature in Pneumonia.**

A patient with pneumonia whose temperature is never above 100° F. usually does badly.—*Dr. Hale White.*

**Bed-Sores.**

To prevent bed-sores the buttocks and back should be carefully washed with soap and water at least twice a day, then methylated spirit or eau-de-Cologne should be freely applied, and afterwards the surface should be dusted with some soothing absorbent powder. A mixture of equal parts of powdered starch, boric acid, and zinc oxide is suitable.

